# Supplementary material for: Immunomic longitudinal profiling of the NeoPembrOv trial identifies drivers of immunoresistance in high-grade ovarian carcinoma
Source: Nat Commun. 2024 Jul 16;15:5932. doi: 10.1038/s41467-024-47000-5 (PMC11252308; doi:10.1038/s41467-024-47000-5)
Supplement: Supplementary file 1 — Supplementary Information [file 41467_2024_47000_MOESM1_ESM.pdf]

Supplementary information

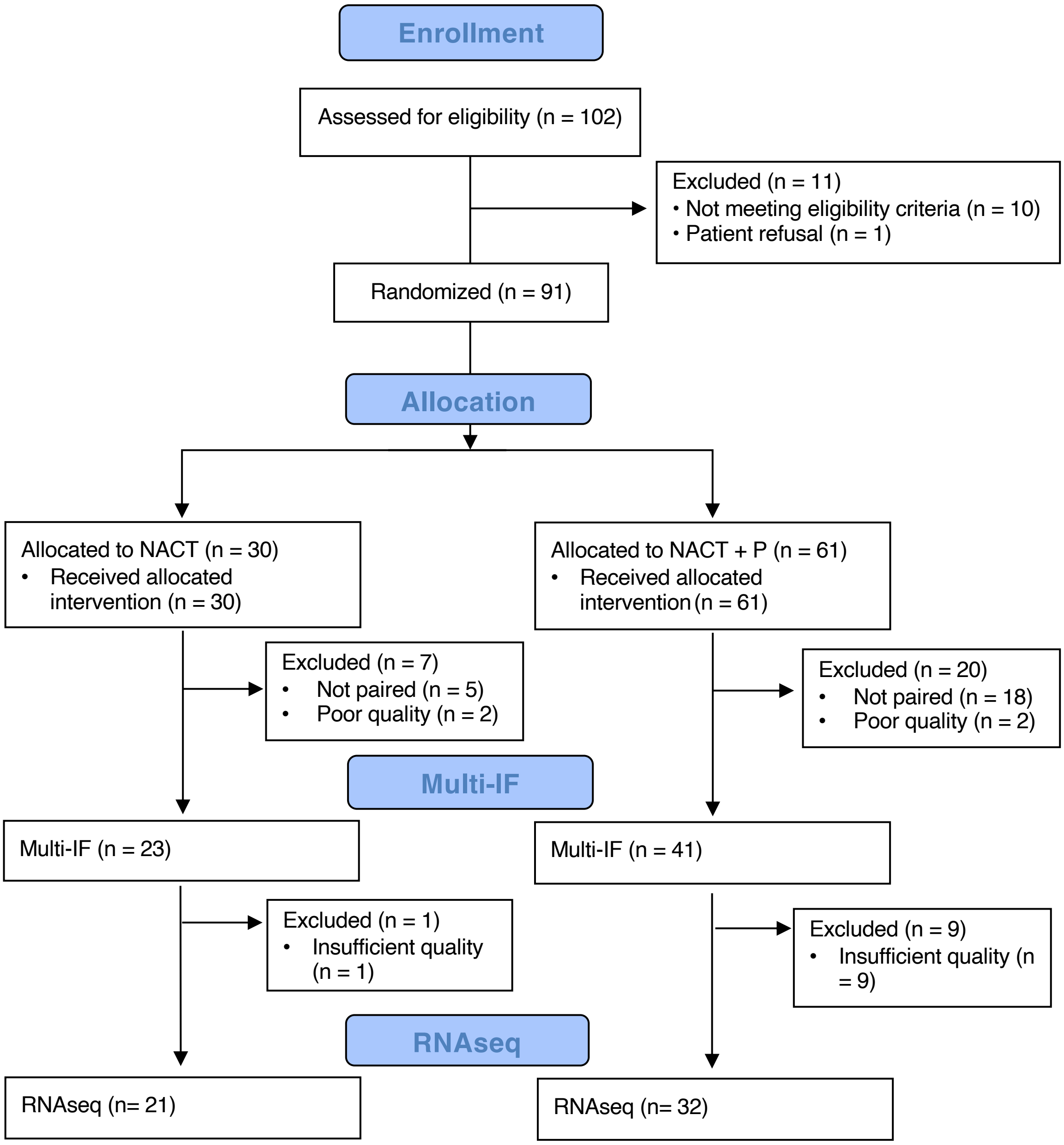

Flow diagram. Flow diagram depicting the number of samples used for each type of analysis in each arm of the clinical trial.

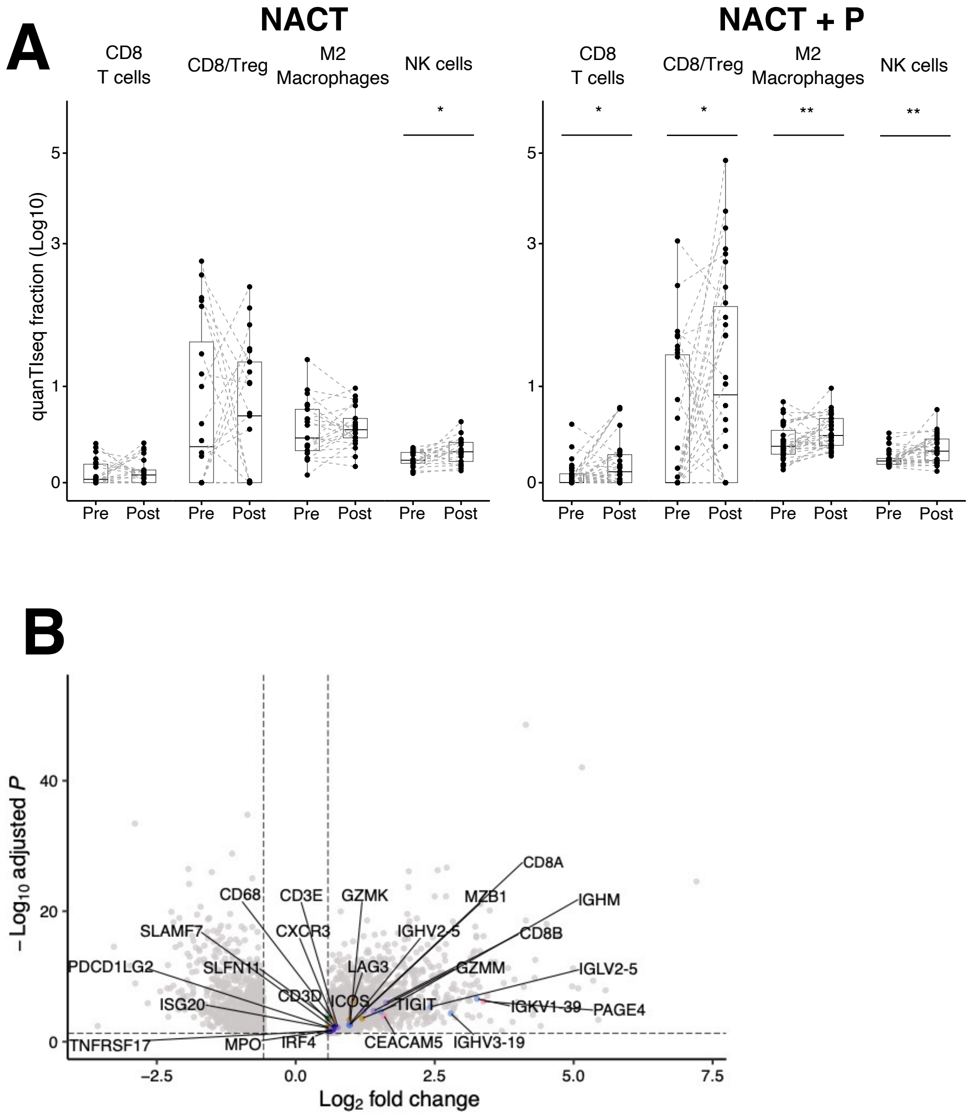

Impact of neoadjuvant chemotherapy +/- Pembrolizumab on the HGSC microenvironment using bulk RNAseq. A. Box and dotplots depicting the quanTIseq gene signature expression scores in pre- and post-treatment samples for each patient in the NACT arm (left, N=21 patients) and the NACT+P arm (right, N=31 patients). The centerline of boxes depicts the median values; the bottom and top box edges correspond to the first and third quartiles. Statistical significance was evaluated using two-sided Wilcoxon signed-rank tests. UnadjP values are represented. NACT: NK cells, unadjP=0.01. NACT+P: CD8 T cells, unadjP=0.031; CD8/Treg, unadjP=0.029; M2 Macrophages, unadjP=0.002; NK cells, unadjP=0.004. **B.** Volcano plot representing the DEG between post- vs pre-treatment samples of patients receiving NACT+P, which were not found in the list of DEG between post- vs pre-treatment samples of patients receiving NACT. Genes with clinical/biological relevance were manually highlighted. Source data are provided as a Source Data file.

Le Saux et al. Supplementary Figure 2

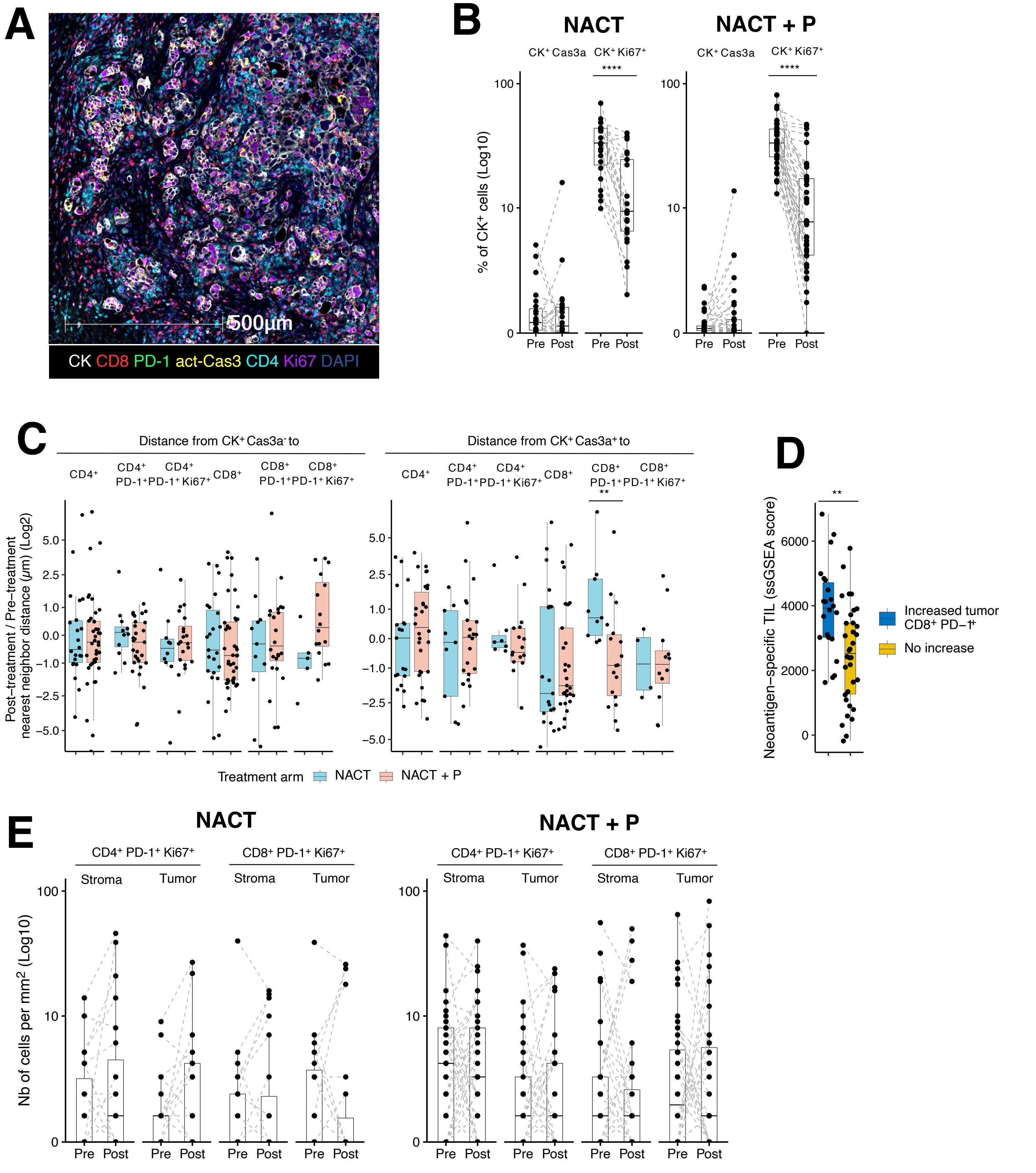

Le Saux et al. Supplementary Figure 3

Evolution of tumor cells and T cells under NACT±P. A. Representative image of the multiplex IF staining focusing on T cell subsets (CD4, CD8) and activation status (PD-1, Ki67), tumor cells (CK) and apoptosis (Act-Cas3a+). **B.** Box and dotplots representing the proportion of Act-Cas3a+ (apoptotic) cells among CK+ (tumor) cells and of Ki67+ (proliferating) cells among CK+ (tumor) cells in NACT arm (left, N=21 patients) and NACT+P arm (right, N=31 patients). The centerline of boxes depicts the median values; the bottom and top box edges correspond to the first and third quartiles. Statistical significance was evaluated using two-sided Wilcoxon signed-rank tests. Unadjusted p-values are showed on the plot for CK+Ki67+ unadjP=4.53e-06 in NACT unadjP=9.09e-13 in NACT+P. **C.** Box and dotplots representing, for each patient, the ratio between the post-treatment and the pre-treatment nearest neighbor distance (μm) from CK+Cas3a- or CK+Cas3a+ cells to CD4+, CD8+, CD4+PD-1+, CD8+PD-1+, CD4+PD-1+Ki67+, and CD8+PD-1+Ki67+ cells in patients receiving NACT alone (blue), N=21 patients, and patients receiving NACT+P (red), N=31 patients. The centerline of boxes depicts the median values; the bottom and top box edges correspond to the first and third quartiles. Statistical significance was evaluated using two-sided Wilcoxon rank sum test. Distance from CK+Cas3a+ to CD8+PD-1+, unadjP=6.25e-03. **D.** Neoantigen-specific tumor infiltrating lymphocyte scores25 showing tumors with (N=22, blue) or without (N=34, yellow) an increase in PD-1+ CD8+ lymphocytes after treatment. The centerline of boxes depicts the median values; the bottom and top box edges correspond to the first and third quartiles. Statistical significance was evaluated using two-sided Wilcoxon rank sum test and unadjP=1.78e-03 is displayed. **E.** Box and dot plots representing the number of cells/mm2 (i.e., the density) of CD4+PD-1+Ki67+ and CD8+PD-1+Ki67+ cells both in the tumor and stroma pre- and post-treatment in the NACT arm (left, N=21 patients) and the NACT+P arm (right, N=31 patients). The centerline of boxes depicts the median values; the bottom and top box edges correspond to the first and third quartiles. Statistical significance was evaluated using two-sided Wilcoxon signed-rank test. Source data are provided as a Source Data file.

Le Saux et al. Supplementary Figure 3

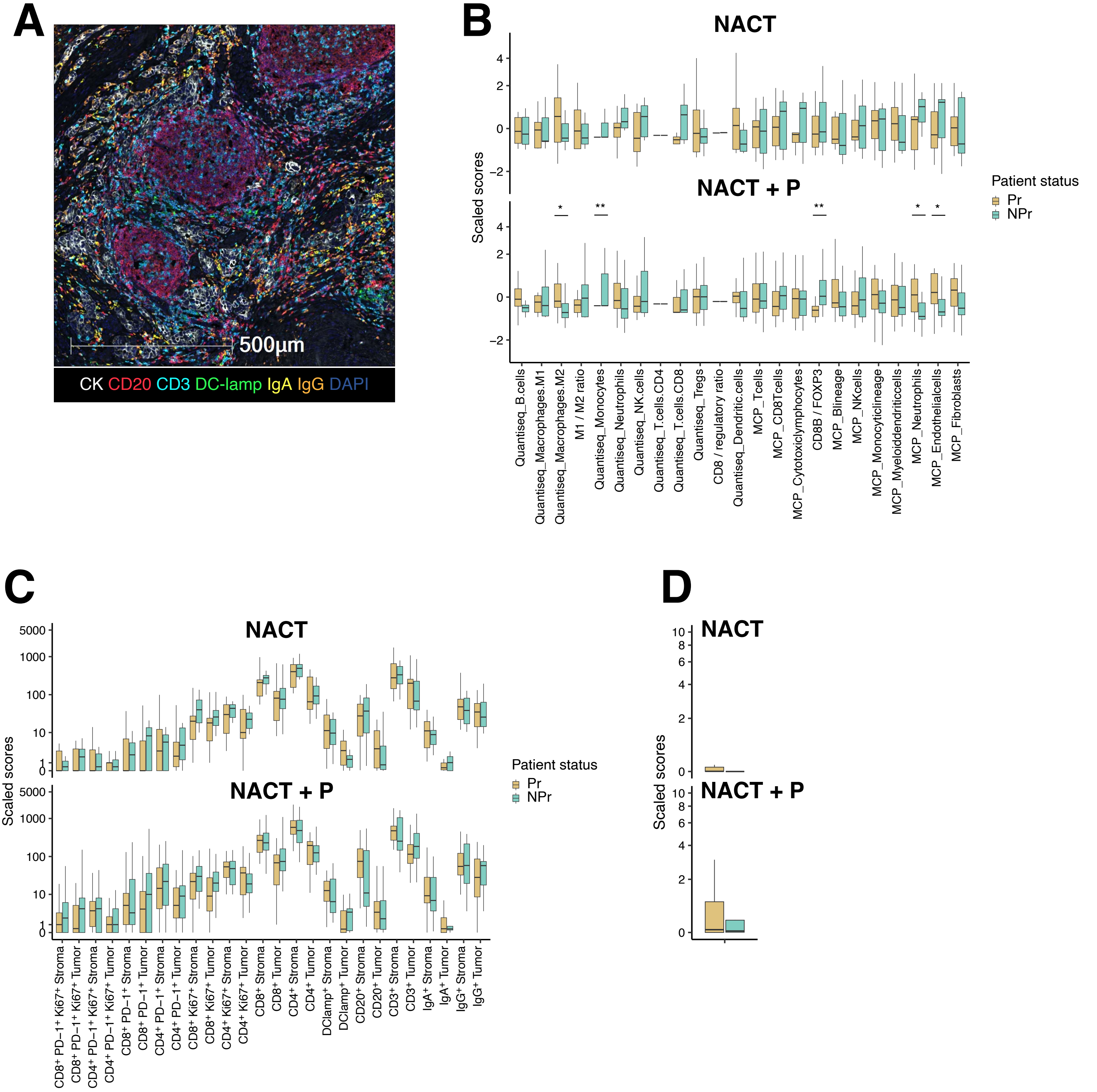

Predictive biomarkers of response to NACT + Pembrolizumab. A. Representative image of the multiplex IF staining focusing on B cells, tertiary lymphoid structures and antibody-secreting cells. **B.C.D.** Boxplots representing the scaled expression scores of various transcriptomic signatures from MCP-counter and quanTIseq (B), the densities of various immune cell subsets in the tumor versus stroma (C) and the % of tumor surface occupied by TLS (D), in non progressors, NPr, (green-blue, N=7 NACT and N=13 NACT+P) versus progressors, Pr, (dark yellow, N=13 NACT and N=19 NACT+P) from NACT arm (top) vs NACT+P arm (bottom). The centerline of boxes depicts the median values; the bottom and top box edges correspond to the first and third quartiles. Significant differences using the two-sided Wilcoxon rank sum test are highlighted with stars on the top (unadjP). Unadjusted p-values for NACT+P displayed on B, QuanTIseq Macrophages M2=0.037; Quantiseq Monocytes=0.006; CD8B/FOXP3=0.008; MCP Neutrophils=0.045; MCP Endothelial cells=0.027. Source data are provided as a Source Data file.

Le Saux et al. Supplementary Figure 4

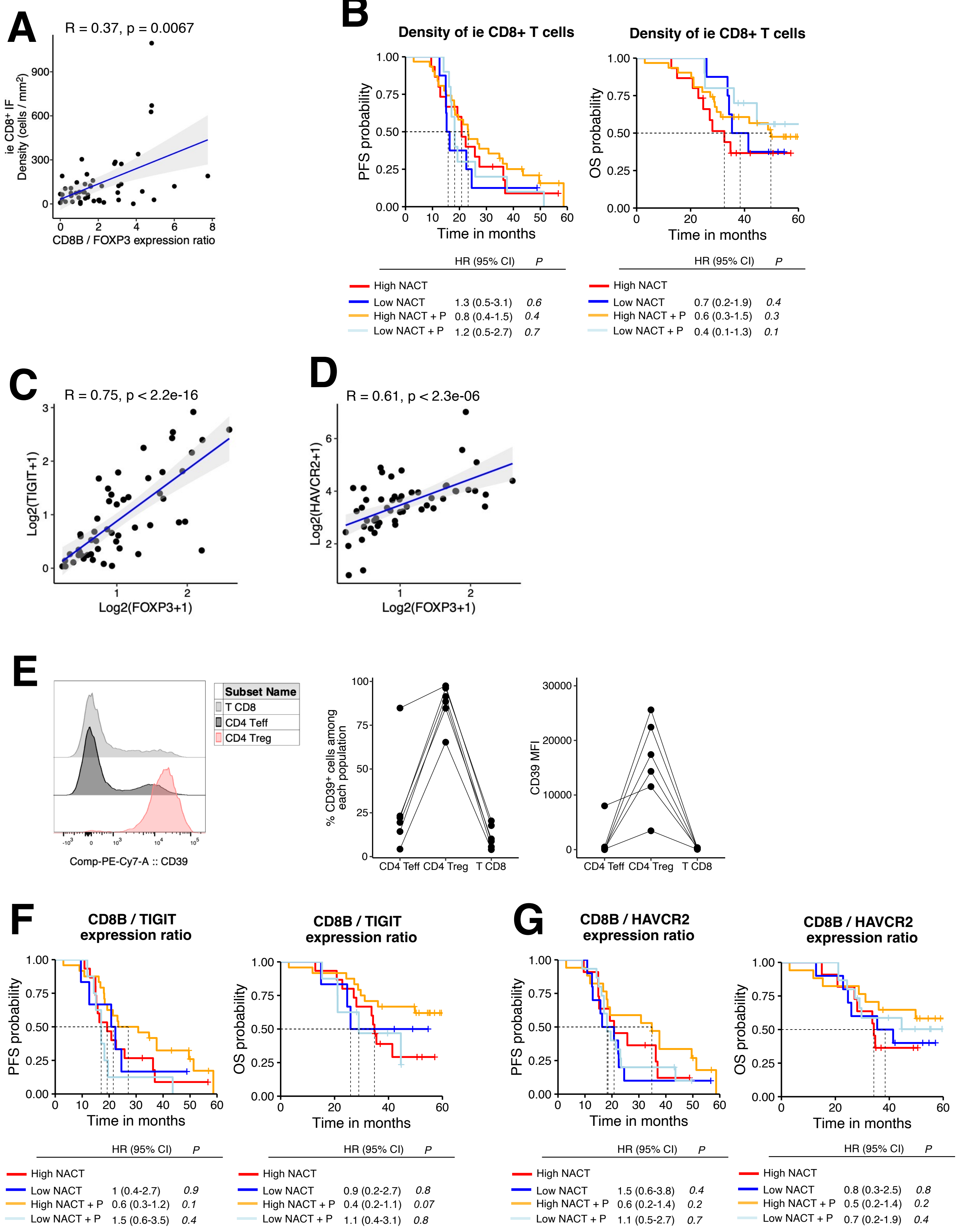

Le Saux et al. Supplementary Figure 5

A high CD8B/FOXP3 gene expression ratio is associated with response to NACT+P. A. Spearman correlation between density of intra-epithelial CD8+ T cells and CD8B/FOXP3 gene expression ratio. Error bands represents the 95% CI as a shaded gray area. **B.F.G.** PFS (left) and OS (right) curves according to the density of intra-epithelial (ie) CD8+ T cells before treatment (B), the CD8B/TIGIT gene expression ratio (High NACT (red), n=15; Low NACT (dark blue), n=6; High NACT+P (orange), n=24; Low NACT+P (light blue), n=8). (F) and the CD8B/HAVCR2 gene expression ratio (High NACT (red), n=11; Low NACT (dark blue), n=10; High NACT+P (orange), n=17; Low NACT+P (light blue), n=15) (G). Patients were stratified based on the best cutoff. Statistical comparison of survival curves for NACT+P High vs. NACT High was performed using the likelihood ratio test. **C.D.** Spearman correlation between log2(TPM+1) FOXP3 expression and TIGIT expression (C) and HAVCR2 expression (D). Error bands represents the 95% CI as a shaded gray area. **E.** Flow cytometry analysis of CD39 expression in CD8 T cells (gated as CD3+CD8+ cells, gray), CD4 Teff (CD3+CD4+Foxp3- cells, black) and CD4 Treg (CD3+CD4+Foxp3+ cells, red) infiltrating ovarian tumors. Histograms of a representative patients (left) and graphs depicting frequency of CD39+ cells and CD39 mean fluorescence intensity (mfi) in the different cell subsets of 6 individual patients (right). Source data are provided as a Source Data file.

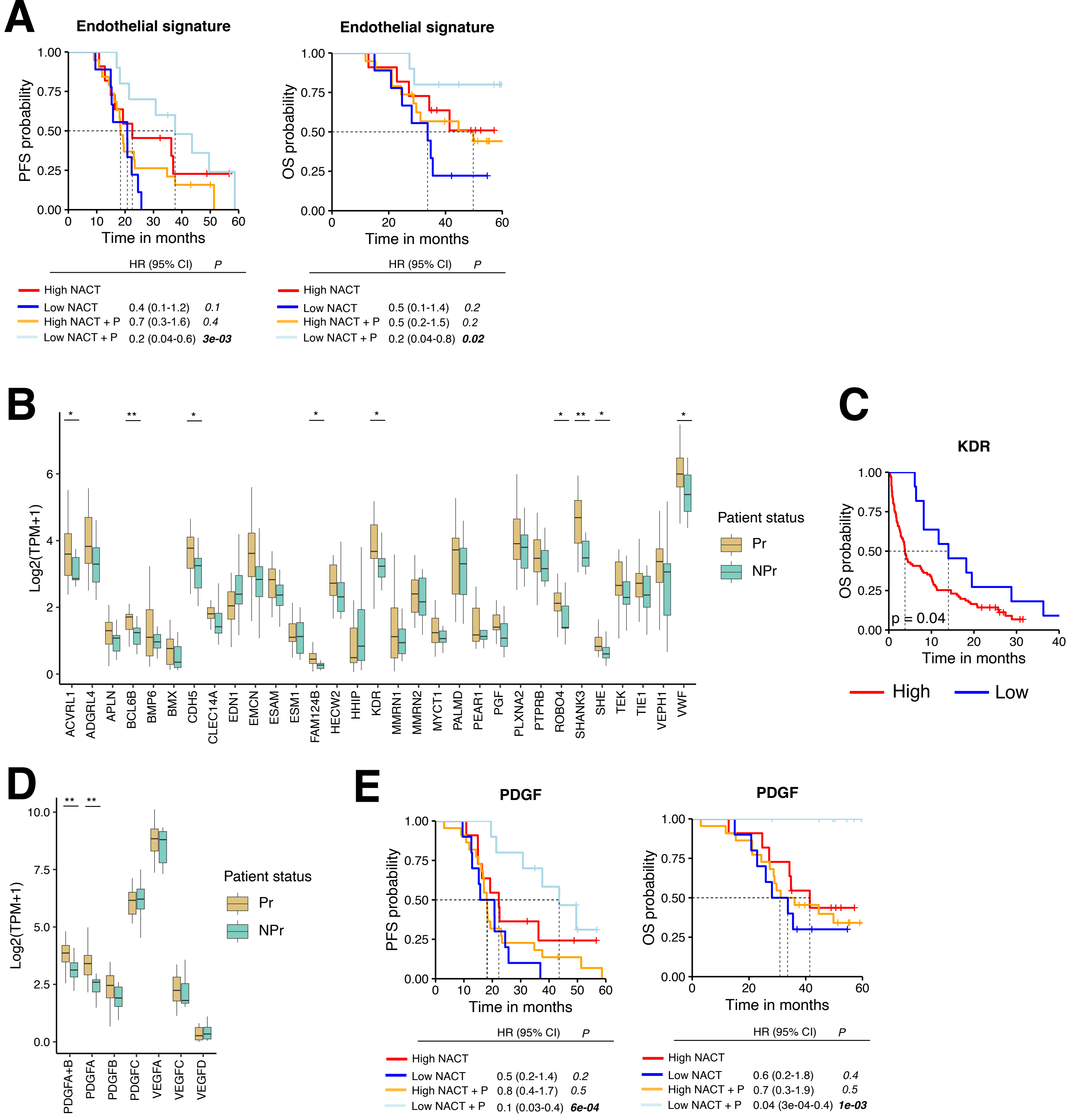

Le Saux et al. Supplementary Figure 6

High expression of KDR/VEGFR2 is associated with resistance to NACT+P. A. PFS (left) and OS (right) curves of patients that received Bevacizumab included in the NeoPembrOv trial according to the expression of the endothelial gene signature (High NACT (red), n=11; Low NACT (dark blue), n=9; High NACT+P (orange), n=19; Low NACT+P (light blue), n=10). Patients were stratified based on the best cutoff. Statistical comparison of survival curves for NACT+P Low vs. NACT Low was performed using the likelihood ratio test. **B.** Association between the expression in TPM of genes included in the MCP endothelial cell signature and patient status (progressors (Pr) in dark yellow vs non progressors (NPr) in green-blue). The centerline of boxes depicts the median values; the bottom and top box edges correspond to the first and third quartiles. Significant differences using the two-sided Wilcoxon rank sum test are highlighted with stars on the top (unadjP). Unadjusted p-values for ACVRL1=0.033; BCL6B=0.007; CDH5=0.041; FAM124B=0.014; KDR=0.0445; ROBO4=0.024; SHANK3=0.002; SHE=0.011; VWF=0.045. **C.** OS curves according to KDR gene expression (High (red), n=91; Low (blue) n=11). **D.** Association between the expression in TPM of genes encoding for VEGFR2/KDR ligands and patient status (progressors (Pr) in dark yellow vs non progressors (NPr) in green-blue). The centerline of boxes depicts the median values; the bottom and top box edges correspond to the first and third quartiles. Significant differences using the two-sided Wilcoxon rank sum test are highlighted with stars on the top (unadjP). **E.** PFS (left) and OS (right) curves according to PDGFA gene expression (High NACT (red), n=11; Low NACT (dark blue), n=10; High NACT+P (orange), n=22; Low NACT+P (light blue), n=10). Patients were stratified based on the best cutoff. Statistical comparison of survival curves for NACT+P Low vs. NACT Low was performed using the likelihood ratio test. Source data are provided as a Source Data file.

Le Saux et al. Supplementary Figure 6

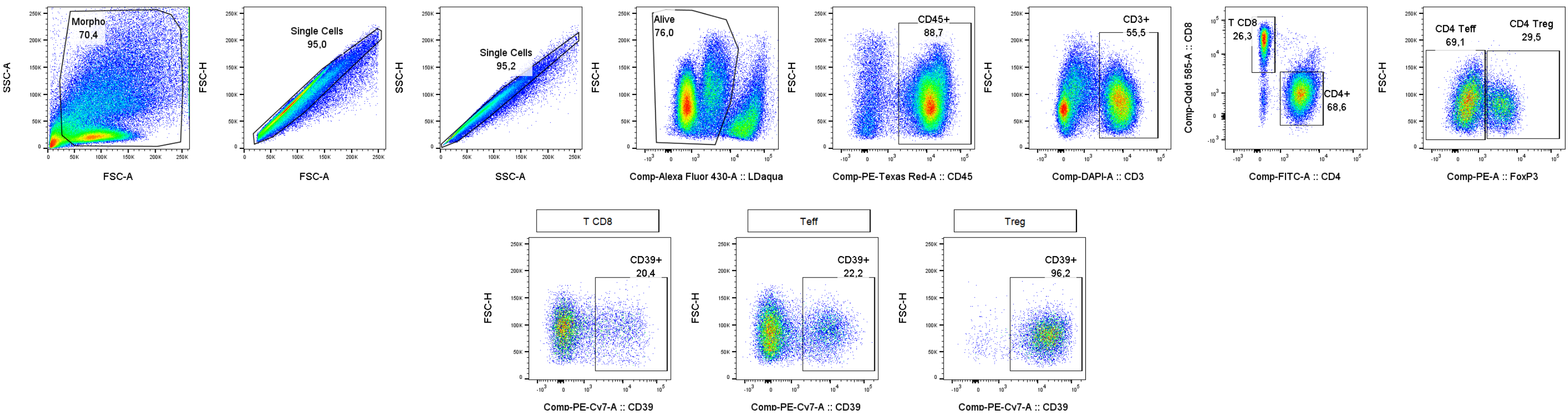

Gating strategy for determination of CD39 expression on T cell populations in human ovarian cancer. After the exclusion of debris, doublets cells (by FSC/SSC plots) and dead cells (LDAqua$^+$), immune cells were selected on CD45 expression (CD45$^+$). The different populations of T cells were gated by the expression of CD8 and CD4 and Teff and Treg were distinguished based on FOXP3 expression (FOXP3^{neg} = Teff and FOXP3$^+$ = Treg). Then CD39 expression was analyzed on each population. Percentage refer to the proportion of cells in the previous parent gate.

Le Saux et al. Supplementary Figure 7

	Total	mIF	p	RNA	p
	N=91	N=64		N=53	
Treatment Arm			0.832		0.532
NACT	30 (33.0%)	23 (35.9%)		21 (39.6%)	
NACT + P	61 (67.0%)	41 (64.1%)		32 (60.4%)	
Age	62.1 (8.83)	61.8 (8.87)	0.858	62.1 (8.83)	0.627
FIGO Stage			1.00		1.000
IIIC	75 (82.4%)	53 (82.8%)		44 (83.0%)	
IV	16 (17.6%)	11 (17.2%)		9 (17.0%)	
BRCA:			1.00		0.880
mutant	19 (21.8%)	14 (22.2%)		10 (19.2%)	
WT	68 (78.2%)	49 (77.8%)		42 (80.8%)	
CC-score:			0.6		0.726
CC-0	66 (75.9%)	53 (82.8%)		44 (83.0%)	
CC-1	2 (2.30%)	1 (1.56%)		1 (1.89%)	
CC-3	19 (21.8%)	10 (15.6%)		8 (15.1%)	
Progression < 2y			1.00		0.928
Yes	54 (60.0%)	39 (60.9%)		33 (62.3%)	
No	36 (40.0%)	25 (39.1%)		20 (37.7%)	

Patient characteristics.

BRCA: Breast Cancer gene, CC-score: Completeness of Cytoreduction score, FIGO: The International Federation of Gynecology and Obstetrics, NACT: NeoAdjuvant ChemoTherapy, P: Pembrolizumab,

Genes	M1-like macrophage (TPM)	M2-like macrophage (TPM)	Monocyte (TPM)	Log2 FC (M2-Like *vs* Monocyte)	Log2 FC (M2-Like *vs* M1-like)
MAOA	5.956	80.147	0.103	9.602	3.750
SLC7A8	12.057	87.139	0.293	8.218	2.853
TREM2	25.083	204.085	0.720	8.148	3.024
NUPR1	101.322	22.433	0.088	7.990	-2.175
MATK	65.493	45.449	0.305	7.220	-0.527

Top 5 genes differentiating monocytes and type 2-like macrophages in quanTIseq. TIL10 signature matrix showing the top 5 genes with the higher log2 fold change in expression between monocytes and type 2-like macrophages.

Genes	M1-like macrophage (TPM)	M2-like macrophage (TPM)	Monocyte (TPM)	Log2 FC (Monocyte *vs* M2-Like)	Log2 FC (M2-Like *vs* M1-like)
CD36	21.986	124.550	457.723	1.878	2.502
CSTA	151.612	99.679	417.400	2.066	-0.605
LILRA2	90.303	8.0705	288.440	5.159	-3.484
NRGN	7.557	31.932	232.978	2.867	2.079
SMARCD3	15.607	10.643	152.812	3.844	-0.552

Top 5 genes expressed in monocytes.

TIL10 signature matrix showing the top 5 genes with the higher expression in monocytes and positive log2 fold change compared to M2-like macrophages.

variable	group1	group2	n1	n2	statistic	p	p.signif	FDR	95CI
CD8B/CTLA4	NPr	Pr	13	19	133	0,734	ns	0,734	[-0.953 - 1.713]
CD8B/ENTPD1	NPr	Pr	13	19	172	0,065	ns	0,195	[-0.005 - 0.076]
CD8B/FOXP3	NPr	Pr	13	19	192	0,008	**	0,084	[0.31 - 2.097]
CD8B/HAVCR2	NPr	Pr	13	19	171	0,071	ns	0,195	[-0.003 - 0.172]
CD8B/ICOS	NPr	Pr	13	19	156	0,223	ns	0,291	[-0.368 - 1.376]
CD8B/ITGAE	NPr	Pr	13	19	166	0,108	ns	0,238	[-0.011 - 0.162]
CD8B/LAG3	NPr	Pr	13	19	160	0,17	ns	0,291	[-0.088 - 0.393]
CD8B/NT5E	NPr	Pr	13	19	155	0,238	ns	0,291	[-0.012 - 0.351]
CD8B/TIGIT	NPr	Pr	13	19	171	0,071	ns	0,195	[-0.034 - 2.407]
CD8B/TNFRSF18	NPr	Pr	13	19	146	0,404	ns	0,444	[-0.803 - 2.14]
CD8B/TNFRSF4	NPr	Pr	13	19	156	0,223	ns	0,291	[-0.438 - 2.223]

Association of selected gene expression ratios with response to the combination of NACT+P.
Two-sided Wilcoxon rank sum test unadjusted p-values without correction for multiple testing.

T panel							
**Marker**	**Clone**	**Provider**	**Reference**	**Species**	**Antigen retrieval treatment**	**Primary antibody incubation time**	**OPAL fluorophore associated**
CD4	EP204	Sigma	104R	Rabbit	ER2 20min	30min	OPAL 570
Ki67	MIB-1	DAKO	M7240	Mouse	ER1 20min	30min	OPAL 690
Cas3 activated	Asp175	Cell Signaling	9661	Rabbit	ER1 30min	30min	OPAL 520
PD1	EPR4877(2)	Abcam	ab137132	Rabbit	ER1 30min	30min	OPAL 780
CD8	C8/144B	DAKO	M7103	Mouse	ER2 20min	30min	OPAL 620
panCK	AE1/AE3	DAKO	M3515	Mouse	ER2 20min	30min	OPAL 480

TLS panel							
**Marker**	**Clone**	**Provider**	**Reference**	**Species**	**Antigen retrieval treatment**	**Primary antibody incubation time**	**OPAL fluorophore associated**
CD3	PC	DAKO	A0452	Rabbit	ER1 20min	30min	OPAL 780
CD20	L26	DAKO	M0755	Mouse	ER1 30min	30min	OPAL 690
DC-Lamp	1010E1.01	Dendritics	DDXO191P	Rat	ER2 20min	30min	OPAL 520
IgA	PC	DAKO	A0262	Rabbit	ER1 20min	30min	OPAL 570
IgG	PC	DAKO	A0423	Rabbit	ER1 20min	30min	OPAL 620
panCK	AE1/AE3	DAKO	M3515	Mouse	ER2 20min	30min	OPAL 480

VEGFR2 panel							
**Marker**	**Clone**	**Provider**	**Reference**	**Species**	**Antigen retrieval treatment**	**Primary antibody incubation time**	**Fluorophore associated**
VEGFR2	B.309.4	Invitrogen	MA5-15157	Rabbit	pH6 60min	60min	Cy3
CD8	4B11	Biorad	MCA1817	Mouse IgG2b	pH6 60min	60min	AF488
CD31	JC70A	DAKO	M0826	Mouse IgG1	pH6 60min	60min	AF647
HEV	MECA79	Biolegend	120804	Rat	pH6 60min	60min	AF594

Antibodies used in multi-IF panels and staining conditions.
