# Supplementary material for: Immunomic longitudinal profiling of the NeoPembrOv trial identifies drivers of immunoresistance in high-grade ovarian carcinoma
Source: Nat Commun. 2024 Jul 16;15:5932. doi: 10.1038/s41467-024-47000-5 (PMC11252308; doi:10.1038/s41467-024-47000-5)
Supplement: Supplementary file 2 — Reporting Summary [file 41467_2024_47000_MOESM2_ESM.pdf]

nature portfolio

Corresponding author(s): Ray-Coquard Isabelle and Dubois Bertrand

Last updated by author(s): Feb 19, 2024

Reporting Summary

Nature Portfolio wishes to improve the reproducibility of the work that we publish. This form provides structure for consistency and transparency in reporting. For further information on Nature Portfolio policies, see our Editorial Policies and the Editorial Policy Checklist.

Statistics

For all statistical analyses, confirm that the following items are present in the figure legend, table legend, main text, or Methods section.

n/a	Confirmed	
☐	☒	The exact sample size (*n*) for each experimental group/condition, given as a discrete number and unit of measurement
☐	☒	A statement on whether measurements were taken from distinct samples or whether the same sample was measured repeatedly
☐	☒	The statistical test(s) used AND whether they are one- or two-sided<br>*Only common tests should be described solely by name; describe more complex techniques in the Methods section.*
☐	☒	A description of all covariates tested
☐	☒	A description of any assumptions or corrections, such as tests of normality and adjustment for multiple comparisons
☐	☒	A full description of the statistical parameters including central tendency (e.g. means) or other basic estimates (e.g. regression coefficient) AND variation (e.g. standard deviation) or associated estimates of uncertainty (e.g. confidence intervals)
☐	☒	For null hypothesis testing, the test statistic (e.g. *F*, *t*, *r*) with confidence intervals, effect sizes, degrees of freedom and *P* value noted<br>*Give P values as exact values whenever suitable.*
☒	☐	For Bayesian analysis, information on the choice of priors and Markov chain Monte Carlo settings
☐	☒	For hierarchical and complex designs, identification of the appropriate level for tests and full reporting of outcomes
☐	☒	Estimates of effect sizes (e.g. Cohen's *d*, Pearson's *r*), indicating how they were calculated

Our web collection on statistics for biologists contains articles on many of the points above.

Software and code

Policy information about availability of computer code

Data collection	For clinical data, Ennov Clinical version v8.2.50. All experimental data were collected and generated as detailed in the Methods section.
Data analysis	For Multi- IF: Vectra Polaris v1.0, Inform v2.5.<br><br>All codes used for analysis in this study were based on the following R packages:<br>DESeq2 v1.28.1 (10.1186/s13059-014-0550-8)<br>ClusterProfiler v3.16.1 (10.1089/omi.2011.0118)<br>ggplot2 v3.3.2 (https://ggplot2.tidyverse.org)<br>ComplexHeatmap v2.4.3 (https://github.com/jokergoo/ComplexHeatmap)<br>glmnet v4.0-2 (doi:10.18637/jss.v033.i01)<br>ROCR v1.0-11 (http://rocr.bioinf.mpi-sb.mpg.de)<br>pROC v1.16.2 (doi.org/10.1186/1471-2105-12-77)<br>survival v3.2-7 (https://CRAN.R-project.org/package=survival)<br>survminer v0.4.9 (https://CRAN.R-project.org/package=survminer)<br>rstatix v0.7.2 (https://CRAN.R-project.org/package=rstatix)<br>phenoptr v0.3.2 (https://akoyabio.github.io/phenoptr/)<br>phenoptrReports v0.3.2 (https://akoyabio.github.io/phenoptrReports/)<br>Coxph R package v1.13.4<br>All bioinformatic tools and methods used in this manuscript have been published previously and no custom code was used.

For further information please contact the corresponding author.
Source data are provided with this paper.

For manuscripts utilizing custom algorithms or software that are central to the research but not yet described in published literature, software must be made available to editors and reviewers. We strongly encourage code deposition in a community repository (e.g. GitHub). See the Nature Portfolio guidelines for submitting code & software for further information.

Data

Policy information about availability of data

All manuscripts must include a data availability statement. This statement should provide the following information, where applicable:

- Accession codes, unique identifiers, or web links for publicly available datasets
- A description of any restrictions on data availability
- For clinical datasets or third party data, please ensure that the statement adheres to our policy

Processed RNA sequencing data presented in the manuscript has been deposited in the Gene Expression Omnibus (GEO) database under the following GEO ID: GSE227666. The processed data are publicly available, https://www.ncbi.nlm.nih.gov/geo/query/acc.cgi?acc=GSE227666. The remaining data are available within the article source data file (ncomms-23-08444B-source-data.xlsx).Raw data access can be asked upon reasonable request.

Processed data corresponds to reads aligned to the reference genome to obtain the gene expression matrix. To ensure samples anonymisation, different IDs are used between expression matrix and patients informations available in supplementary data.
Data sharing in a public repository was not planned at the start of the study. Per European and French regulations for personal data privacy, this is not permitted without having informed the study participants which was not done. This is also linked to a confidentiality agreement with MSD who provided the drug and funding. This agreement aims to guarantee protection for the company about potential sub-licensable or patentable information/discovery. Requests to access the deidentified data for further scientific use can be sent to ARCAGY-GINECO (Sébastien Armanet sarmanet@arcagy.org) will be considered on a case-by-case basis in a timely manner beginning 3 months and ending 5 years after this article publication. Request must contain a proposal with scientific and methodologically justified objectives. A Data Transfer Agreement will be established to provide a formal framework regarding the use of the data.

Research involving human participants, their data, or biological material

Policy information about studies with human participants or human data. See also policy information about sex, gender (identity/presentation), and sexual orientation and race, ethnicity and racism.

Reporting on sex and gender	Findings only apply to one sex or gender (ovarian cancer study).
Reporting on race, ethnicity, or other socially relevant groupings	Information on race and ethnicity was not collected in this relatively small single-country study.
Population characteristics	Population characteristics are described in supplementary table 1.
Recruitment	Patients were recruited by the investigators according to the inclusion/exclusion criteria of the study protocol (no recruitment by internet, no recruitment by advertisement). There was no compensation for participation.
Ethics oversight	CPP Nord Ouest II

Note that full information on the approval of the study protocol must also be provided in the manuscript.

Field-specific reporting

Please select the one below that is the best fit for your research. If you are not sure, read the appropriate sections before making your selection.

[x] Life sciences [ ] Behavioural & social sciences [ ] Ecological, evolutionary & environmental sciences

For a reference copy of the document with all sections, see nature.com/documents/nr-reporting-summary-flat.pdf

Life sciences study design

All studies must disclose on these points even when the disclosure is negative.

Sample size	Assuming a 50% CRR at IDS with NACT alone, as reported in the literature, the planned sample size of 90 patients (60 in the experimental investigational arm, 30 in the standard-of-care control arm) was calculated based on the A'Hern single-stage design,36 with a ≥70% success rate (CC0 at IDS) in the pembrolizumab arm considered sufficient to justify further evaluation and a <50% rate considered insufficient.
Data exclusions	For translational analyses, analyses were performed on all patients with available paired samples and sufficient quality as shown in supplementary figure 1.
Replication	Due to tissue availability concerns, RNA extractionand sequencing and multi-IF stainings were not replicated.
Randomization	Patients were randomly allocated to NACT or NACT+P.
Blinding	Investigators were not blinded to the administration of Pembrolizumab as it was a randomized non comparative phase II study. It was a

seeking signal phase II trial not a pivotal phase III trial necessitating blinding to limit any bias.

Reporting for specific materials, systems and methods

We require information from authors about some types of materials, experimental systems and methods used in many studies. Here, indicate whether each material, system or method listed is relevant to your study. If you are not sure if a list item applies to your research, read the appropriate section before selecting a response.

Materials & experimental systems

n/a	Involved in the study
[ ]	[x] Antibodies
[x]	[ ] Eukaryotic cell lines
[x]	[ ] Palaeontology and archaeology
[x]	[ ] Animals and other organisms
[ ]	[x] Clinical data
[x]	[ ] Dual use research of concern
[x]	[ ] Plants

Methods

n/a	Involved in the study
[x]	[ ] ChIP-seq
[ ]	[x] Flow cytometry
[x]	[ ] MRI-based neuroimaging

Antibodies

Antibodies used

For multi-IF: (supplementary table 5)
*T panel
CD4 EP204 Sigma 104R
Ki67 MIB-1 DAKO M7240
Cas3 activated Asp175 Cell Signaling 9661
PD1 EPR4877(2) Abcam ab137132
CD8 C8/144B DAKO M7103
panCK AE1/AE3 DAKO M3515

*TLS panel
CD3 PC DAKO A0452
CD20 L26 DAKO M0755
DC-Lamp 1010E1.01 Dendritics DDXO191P
IgA PC DAKO A0262
IgG PC DAKO A0423
panCK AE1/AE3 DAKO M3515

*VEGFR2 panel
VEGFR2 B.309.4 Invitrogen MA5-15157
CD8 4B11 Biorad MCA1817
CD31 JC70A DAKO M0826
HEV MECA79 Biolegend 120804

For FCM (supplementary table 6)
CD4 FITC 555346 BD
FoxP3 PE 12-4776-42 eBioscience
CD45RO PerCP Cy5.5 560607 BD
CD39 PECy7 25-0399-42 eBioscience
CD3 BV421 300434 Biolegend
Zombie Aqua 423102 Biolegend
CD45RA ECD B49193 Beckman Coulter
CD8 BV570 301038 Biolegend

Validation

Blood for FCM and IHC for multi-IF as available on the manufacturer's websites.

Clinical data

Policy information about clinical studies

All manuscripts should comply with the ICMJE guidelines for publication of clinical research and a completed CONSORT checklist must be included with all submissions.

Clinical trial registration	Clinicaltrials.gov number NCT03275506
Study protocol	The protocol is provided in the supplementary materials of the clinical paper.
Data collection	Between February 26, 2018, and April 17, 2019, 91 patients were enrolled from 17 sites in France. Data were collected in academic centers in an electronic case report form (CS Online Ennov Clinical version v8.2.50 powered by Euraxi Pharma, a French contract research organization). The data were monitored through on-site monitoring visits by clinical research associates according to a prespecified monitoring plan. All data were centralized in a database that was handled and controlled according to a specific data management plan, and analyzed using SAS version 9.4
Outcomes	The primary endpoint was CRR, defined as the removal of all macroscopic residual tumor (CCI = 0; CC0) at IDS, as assessed by blinded independent centralized review by two surgical experts and the coordinating investigator, who reviewed the anonymized operative and pathologic reports of all patients at screening, at IDS, and at other debulking surgery.<br>Secondary efficacy endpoints included CCI score by local assessment, PCI score by local and central assessment (to be reported separately), ORR after four neoadjuvant cycles according to RECIST version 1.1, best overall response to the global strategy, PFS according to RECIST version 1.1 (defined as the interval between randomization and date of disease progression or death, whichever occurred first), and OS. Other secondary endpoints included safety during NACT and in the adjuvant setting, postoperative mortality, and postoperative morbidity according to modified Clavien-Dindo classification.

Plants

Seed stocks	NA
Novel plant genotypes	NA
Authentication	NA

Flow Cytometry

Plots

Confirm that:

- [x] The axis labels state the marker and fluorochrome used (e.g. CD4-FITC).
- [x] The axis scales are clearly visible. Include numbers along axes only for bottom left plot of group (a 'group' is an analysis of identical markers).
- [x] All plots are contour plots with outliers or pseudocolor plots.
- [ ] A numerical value for number of cells or percentage (with statistics) is provided.

Methodology

Sample preparation	Tumor samples were mechanically dilacerated in RPMI-1640, according to a standard calibration (2 mL/g of tumor). Tumor dilacerates were then enzymatically digested for 45 min at 37°C using Collagenase-IV (200U/mL; Invitrogen) and DNase-I (25 µg/mL; Merck) in RPMI-1640. Dilacerates were filtered on 70 µm strainer to obtain TME single-cell suspension.<br>The FCM panel used to assess T-cell differentiation relied on the use of anti-human antibodies against CD45, CD3, CD4, CD8, FOXP3, CD39, and a viability marker (Supplementary Table 6). Cell suspension were first stained with Zombie Aqua (Cat: 423102, Biolegend) to remove dead cells. For surface staining, cells were stained with fluorochrome-labelled antibodies in the staining buffer (PBS with 5% FBS, 2mM EDTA) at 4°C for 30 min. Cells were then fixed with 2% formaldehyde (Merck).
Instrument	Cells were analyzed on a LSR-Fortessa 4 lasers (BD Biosciences).
Software	Data were processed using the FlowJo 10.6.2 Software (Tree Star).
Cell population abundance	Not applicable
Gating strategy	The lymphocytes were first gated within the lymphocyte region in the FSC/SSC plots, then the doublets were removed by

gating the diagonal cells in the FSC-H vs FSC-A plots. Immune cells were selected by gating on CD45+ cells. The T cells were selected by gating on CD3+ T cells. Based on previous works all cell being memory T cells in ovarian tumor environment, CD4 Teff were defined as CD4+/FOXP3neg, CD4 Treg as CD4+/FOXP3+ and T CD8 as CD8+. CD39 was then assessed on each of these populations.

☒ Tick this box to confirm that a figure exemplifying the gating strategy is provided in the Supplementary Information.
